# Prevention of Filipino Youth Behavioral Health Disparities: Identifying Barriers and Facilitators to Participating in “Incredible Years,” an Evidence-Based Parenting Intervention, Los Angeles, California, 2012

**DOI:** 10.5888/pcd12.150186

**Published:** 2015-10-22

**Authors:** Nicole Flores, Jocelyn Supan, Cary B. Kreutzer, Allan Samson, Dean M. Coffey, Joyce R. Javier

**Affiliations:** Author Affiliations: Nicole Flores, Jocelyn Supan, Cary B. Kreutzer, Allan Samson, Dean M. Coffey, Department of Pediatrics, Division of General Pediatrics, Children’s Hospital Los Angeles, University of Southern California Keck School of Medicine, Los Angeles, California

## Abstract

**Introduction:**

Evidence-based interventions for training parents are proven to prevent onset and escalation of childhood mental health problems. However, participation in such programs is low, especially among hard-to-reach, underserved populations such as Filipino Americans. Filipinos, the largest Asian subgroup in California, have significant behavioral health disparities compared with non-Hispanic whites and other Asian subgroups. The purpose of this study was to learn about Filipinos’ barriers and facilitators to participating in “Incredible Years” (IY), a parenting program.

**Methods:**

We conducted 4 focus groups in Los Angeles, California, in 2012; the groups consisted of 20 Filipino parents of children aged 6 to 12 years who recently completed the IY parenting program, which was offered as a prevention workshop. Three reviewers, including two co-authors (A.S., J.J.) and a research assistant used content analysis to independently code the interview transcripts and extract subthemes. Grounded theory analytic methods were used to analyze interview transcripts.

**Results:**

Parents’ perceived benefits of participation in IY were learning more effective parenting techniques, networking with other parents, improved spousal relationships, and improvements in their children's behavior. Parents’ most common motivating factor for enrollment in IY was to improve their parenting skills and their relationships with their children. The most common barriers to participation were being uncomfortable sharing problems with others and the fear of being stigmatized by others judging their parenting skills. Participants said that parent testimonials would be the most effective way to promote IY. Many recommended outreach at schools, pediatricians’ offices, and churches.

**Conclusion:**

Increasing Filipino American parent enrollment in IY in culturally relevant ways will reduce the incidence of mental health disorders among children in this growing population.

## Introduction

Mental health disorders are widespread among children and are a significant burden for both children and their caregivers. One in 5 children has a diagnosable mental disorder, and the lifetime prevalence of mental disorders among adolescents is nearly 50% (1,2). Despite these numbers, which are increasing, only 20% of children with mental disorders have their disorder diagnosed and receive mental health services (3).

Filipinos are the second largest Asian subgroup in the United States and the largest subgroup in California, numbering 2,649,973 in 2010 and making up 21% of US Asian children (4,5). Filipino youths have a disproportionately heavy burden of behavioral problems, including higher rates of high school drop-out, depression, teen pregnancy, and substance use, compared with whites and other Asian subgroups. However, they are less likely to participate in mental health and preventive care interventions (6) and have significant mental health risk factors, including exposure to harsh parental discipline and parents who have their own high levels of unmet mental health needs (7). Barriers to help-seeking include 1) an intergenerational gap between Filipino parents and children and 2) cultural factors such as Filipinos’ tendency to passively accept the way things are (*bahala na*), to avoid shame (*hiya*) related to accessing mental health services, and to put the needs of family above personal desires (8–11).

Evidence-based parenting interventions prevent youths from developing behavioral health problems (12,13). One such intervention is “Incredible Years” (IY), which focuses on strengthening parent competencies and decreasing children’s problem behaviors. The intervention has been applied in various cultures with programs tailored to different developmental levels (14). Ten randomized controlled trials of IY conducted among diverse populations showed significant improvements in positive parenting interactions and reductions in harsh discipline and children’s aggressive behavior (11). Promising results from IY were obtained after previous studies with other Asian subgroups (15,16). However, participation rates in such interventions are low, especially among hard-to-reach populations, such as Filipinos (8,10). Few studies explored barriers and facilitators to participation in the IY program by Filipinos, a growing population. Information is needed to fill this knowledge gap and thus increase participation in evidence-based parenting interventions and decrease the rate of mental health problems among Filipino youths. Our study objectives were to learn about participation benefits, motivations, and barriers in the IY program by conducting focus groups of IY participants and to develop a marketing strategy to increase participation rates.

## Methods

### Incredible Years eligibility and focus group recruitment

We conducted focus groups of IY study participants who were enrolled in a pilot randomized controlled trial that involved participation in the 12-week IY parent program, pre- and post-surveys, and participation in a focus group on completion of the IY program. The trial took place from October 2011 to August 2012. Participants were randomly assigned to participate either in an immediate 12-week IY parenting intervention program or to a 4-month wait list control condition followed by the intervention. IY participants were primarily recruited from 2 Catholic churches with predominantly Filipino-American congregations and an associated Catholic school. Parenting groups were conducted concurrently with catechism classes to increase participation by parents. The inclusion criteria for participation were being a self-identified Filipino-American parent and the primary caregiver for a child aged 6 to 12 years; the willingness of at least 1 parent to complete the 12-week IY parenting intervention, pre- and post-surveys, and a focus group; and self-reported English language fluency. The exclusion criteria were having a target child with developmental disabilities or the parent’s inability to speak English. The study was approved by the institutional review board of Children’s Hospital Los Angeles, and we obtained written informed consent from all participants.

### Focus groups

Of the 22 parents who completed the IY pilot program, 20 agreed to participate in 1 of 4 focus groups, which were conducted in April 2012 and August 2012. Focus groups were conducted primarily in English, but a Tagalog interpreter was present at each session to provide interpretation if needed. Each focus group had 5 parents, a trained facilitator, a co-facilitator, a bilingual interpreter, and a note taker. The sessions were 90 minutes long, and all sessions were digitally recorded and transcribed.

At each session, the facilitator asked parents questions from the following 4 topic areas related to IY: facilitators of engagement and strategies for increasing future enrollment, obstacles to engagement, ways to motivate other parents to attend this seminar, and cultural and attitudinal barriers. The groups concluded by soliciting additional comments and open discussion from parents**.** Gift cards were given as incentives for participating in the focus groups. Transcripts were analyzed using a methodology of coding consensus, co-occurrence, and comparison described by Wilms et al (17) and rooted in grounded theory, which is derived from data and then illustrated by characteristic examples of data (18) (see the Appendix for focus group questions).

## Results

Most participants self-identified as their child’s biological mother, as married, and with a college education (Table). The majority of parents identified themselves as Filipino only, and the remaining 2 participants self-identified as Filipino–Chinese and White–Caucasian but born in the Philippines.

**Table:** Characteristics of Parents (N = 20) Who Participated in Focus Groups From “Incredible Years,” an Evidence-Based Parent Training Intervention, Los Angeles, California, 2011–2012

Characteristic	Range (Mean [SD])
**Age, y**	31–65 (43.55 [7.52])
**No. of children in the parent participant’s home **	1–4 (1.95 [1.0])
**Characteristic**	**Number**
**Sex**	
Male	2
Female	18
**Relationship to child**	
Biological mother	16
Biological father	2
Adoptive parent	2
**Marital status**	
Living together	1
Married	18
Separated	1
**Child’s sex**	
Male	9
Female	11
**Annual income^a^**	
<$4,999–$29,999	1
$30,000–$34,999	1
$35,000–$39,999	1
$40,000–$44,999	3
$45,000–-$49,999	7
≥$60,000	6
**Education**	
Grade 0–8	1
Grade 9–11	1
Some college	3
College graduate	14
Post-college degree	1
**Employment status**	
Employed full time	12
Employed part time	3
Working at home	1
Not working, looking for a job	4
**Race/ethnicity**	
White or Caucasian	1
Filipino and Chinese	1
Filipino only	18
**Birthplace (Country/region of Philippines or United States)**	
Central Luzon	3
Calabarzon	2
Bicol	1
Western Visaya	1
National Capital Region	11
United States	2

Abbreviation: SD, standard deviation.

a Numbers do not total 20 because one participant refused to answer.

### Benefits and motivation for participation

**Effective parenting.** Parents identified the IY parenting techniques as the most useful benefits they received from participating in the program. Use of praise and encouragement was the most frequently mentioned technique: “[IY] helped me understand that praising [or] rewarding your kids does not necessarily mean that you’re spoiling them. Positive reinforcement will help them later on.” Other popular parenting techniques included time-out, spending special time with their children, and setting rules, responsibilities, and routines.

**Group support.** Many participants benefited from forming new relationships with other parents in the program. They expressed appreciation for the opportunity to learn from others’ experiences, discuss parenting techniques, and rely on other parents for extra support. As one parent described his feelings about group support, “Sometimes you are trying to deal with an issue [and] feel, ‘Why am I the only one?’ But other parents have gone through that, and they have another perspective that you can look at that will help you.”

**Improved child behavior and family relationships.** Several parents observed improvement in their children’s behavior during their participation in the parenting groups. The most commonly reported improvement was in their child’s social skills. One parent described the impact on her daughter’s social skills: “When we started [the group], sometimes it took time for her to socialize. Now I could see her start talking. When I enrolled her into a swimming class, I could see her right away talking to other people, which really helped her be more confident.” Parents also noted improvements in their child’s school performance and ability to learn from their mistakes and follow directions.

Several parents learned different strategies that allowed them to better co-parent with their spouses and reported improved marital relationships after going through the program. Many parents also developed closer relationships and improved communication with their children.

**Motivation to join and satisfaction with program.** The most common motivating factor for parents to enroll in IY was to improve their parenting skills and, ultimately, their relationships with their children. A few parents felt encouraged to join after seeing that the program was adapted for the specific needs of Filipinos and also saw it as a chance to have a positive effect on their community.

Overall, parents felt satisfied with the IY program, stating that it was informative and that the techniques were effective and presented “. . . in an order that’s making you progress from nothing to really something.”

### Barriers to participation

**Discomfort with self-disclosure.** Parents suggested barriers that could discourage other Filipino parents from participating in IY. Most commonly, they felt that Filipino parents would feel uncomfortable sharing their problems with others, especially with those who have a different cultural background. One parent described this feeling of shame poignantly: “All the stuff in their closet, all the dirty laundry they don’t want to air out is going to be out there for the public to see no matter how many consents you sign or disclaimers.”

**Stigma related to perceived incompetence.** Another major barrier mentioned is the stigma that could result from others judging them as incompetent parents. One parent related her own experience of telling a friend about her participation in IY: “I used the word[s], *parenting class*, and [my friend responded,] ‘What’s wrong with you? You don’t need parenting classes to raise your kid!’” Some parents further said that the stigma associated with being a parent of a child with a behavioral problem could dissuade others from participating.

**Cultural beliefs about parenting.** Other parents expressed the opinion that some Asians and Filipinos are not open to new ideas about how to raise their children “because some people would say, ‘I learned this from my parents so what I’m doing is already okay . . . Why do I have to attend a parenting class?’” Furthermore, most parents commented on the difficulties of parenting and disciplining a child in the United States after growing up primarily in an authoritarian household in the Philippines. One parent explained it this way: “I grew up in the Philippines, and of course, it’s always ‘I’m the parent, and you’re the child. I’m the authority.’ They [children] don’t question authority.” Participants also discussed how some parents may deny that their child has a mental health or behavioral issue and instead, explain it as “Oh that kid was just born bad! That’s it. End of the story.”

**Language and other barriers.** Although Tagalog interpreters participated in focus groups, participants expressed the belief that those who were less fluent in English might be hesitant to join and would rather attend classes in their native language.

Cost was also mentioned frequently as a barrier even though the program was free. The program’s name could also influence whether parents would sign up. Some parents recommended avoiding the word “research” in recruitment since it is often perceived negatively and might make parents feel as if they are being used as guinea pigs by researchers.

**Developing a marketing strategy.** Parents offered suggestions about how to market the IY program. A wide majority of parents suggested that the most effective means to promote participation would be testimonials and success stories from parents who are either participating in or have already successfully completed IY. Furthermore, parents believed it would be helpful to focus on specific benefits and examples of what is entailed in various parts of the program instead of simply providing a brief overview. Parents also recommended using teachers, physicians, or pastors as endorsers of the program, none of whom had to be Filipino.

Most participants felt that the target audience should be anyone with children, including coworkers, friends, relatives, and newly immigrated families. They recommended marketing at schools as was done for this study, but some also suggested promoting the program at pediatric clinics, churches, and Filipino retail establishments. Others suggested using a media or video campaign to produce a commercial for the program or advertising in a newspaper to reach as many people as possible.

## Discussion

We learned of benefits to participating in an evidence-based parenting intervention, barriers to participating, and methods of marketing to increase participation in these programs by Filipino-American parents. The most widely acknowledged benefit of participation in IY was learning effective techniques for parenting, which enhanced participants’ relationships with their children and their spouses. This finding is consistent with findings from studies in which parents were asked about the benefits of the program in which they participated (15,19). Reid et al, in particular, addressed the broad applicability of the techniques and topics taught in IY to various cultural beliefs (13). Applicability is especially relevant to our study and target population, because the intervention was targeted to Filipino-American parents, who still hold strongly to their cultural traditions and authoritarian family dynamics. Cultural sensitivity and relevance is a significant factor in the uptake of the program by different cultural groups (19). The other benefits discussed in the focus groups — most notably parental support and improved relationships with their children — are also consistent with previous findings from studies in which parents were directly interviewed after participating in the program (20).

Barriers to participation and continued engagement in IY are active areas of research because of increasing interest in disseminating evidence-based parenting programs, especially among hard-to-reach populations. Koerting et al identified various types of barriers, such as situation barriers (eg, inadequate transportation or childcare) and psychological barriers (eg, stigma, shame, distrust), which are most consistent with our findings in working with this population (20). Stigma is a particularly important factor to consider with regard to the Filipino population because of parents’ shame and perception of failing when they admit they need help (21,22). Shame and fear of stigma create significant barriers to participating in parenting programs.

Another barrier found during the focus groups was the participants’ perception that the intervention was research and therefore could be seen in a negative way: researchers treating the parents simply as guinea pigs. This suspicion and distrust were noted in previous studies and remain important issues that must be addressed in future studies (23–25). A strong therapeutic alliance and transparency must be established and maintained with the Filipino community to lessen these concerns. Of note, parents in our focus groups did not consider prominent barriers mentioned in other studies, such as difficulties with transportation or childcare, (26) likely due in part to our implementation strategy (ie, offering the program while children were attending catechism and offering child care if needed).

The last aim of our study was to learn the most effective means of marketing this program to Filipino-American parents, given their experience with the program and their cultural beliefs about parenting. Consistent with previous studies, we found that first-hand testimonials were seen as the most persuasive means of convincing a parent to enroll in the program (20,27). Findings from our focus groups similarly indicate that our participants believe that the locations where the program is advertised and the modes of advertising are important; participants mentioned multichannel promotion, such as flyers, commercials, and newspaper advertisements in places of importance to the culture (28). One potential facilitator to enrollment that was not previously emphasized is endorsement from respected community members. These endorsers could be teachers, physicians, or pastors, none of whom had to be Filipino. These community endorsements may serve as additional avenues to increase enrollment in evidence-based parenting interventions.

The use of focus groups involves some limitations. First, individual dynamics may vary in a group setting and affect whether a parent contributes to the discussion or relates experiences. The overall tone and collective opinion of the group may vary from group to group, depending on the individual participants in the group.

Second, the generalizability of these findings is limited by the small sample size and by the convenience sampling methods employed in this study. Furthermore, the demographic makeup of our sample was characterized by predominantly Catholic, 2-parent households, and immigrant families residing in the Historic Filipinotown district of Los Angeles, a low middle-class, working neighborhood. Although the majority of Filipinos are immigrants and Catholic (29), our findings may not be generalizable to the broader Filipino population in the United States.

Despite these limitations, findings from this study may inform future efforts to increase Filipino-American participation in evidence-based parenting interventions. Further study is needed to explore how to overcome cultural mistrust among Filipino families. It would be worthwhile to study the impact on engagement and enrollment in evidence-based parenting interventions when culturally sensitive modes of outreach are used.

Parenting interventions can decrease the later incidence of mental health disorders among youths by equipping parents with the tools needed to raise successful and well-adjusted children (12,13). Increasing Filipino-American parent enrollment in IY through culturally relevant means may decrease mental health disorders in this growing population. Further studies are needed to develop and test engagement strategies aimed at increasing participation of Filipino parents in evidence-based parenting interventions.
